# NOE distance and dihedral angle restraints to calculate the solution structure of the NDH-1 complex subunit CupS from *Thermosynechococcus elongatus*

**DOI:** 10.1016/j.dib.2015.12.004

**Published:** 2015-12-15

**Authors:** Annika Korste, Hannes Wulfhorst, Takahisa Ikegami, Marc M. Nowaczyk, Raphael Stoll

**Affiliations:** a Biomolecular Spectroscopy, Faculty of Chemistry and Biochemistry, Ruhr University of Bochum, Bochum, Germany; b Department of Plant Biochemistry, Faculty of Biology, Ruhr University of Bochum, Bochum, Germany; c Institute for Protein Research, Osaka University, Japan

**Keywords:** Bioenergetics/electron Transfer Complex, CupS, Cyanobacteria, Membrane Proteins, NMR Protein Structure, NOE Distance And Dihedral Angle Restraints

## Abstract

Here, we have compiled a nuclear magnetic resonance (NMR)-derived set of nuclear Overhauser enhancement (NOE) distance and dihedral angle restraints that allow for the calculation of the structure of the NDH-1 complex subunit CupS from *Thermosynechococcus elongatus* in solution. These restraints to calculate the structure in solution of CupS have been deposited to the Protein Data Bank (www.rcsb.org) under PDB-ID accession number **2MXA**. This is the first experimental data set published to compute the three-dimensional structure of CupS. This structure is presented in the research article “Solution structure of the NDH-1 complex subunit CupS from *Thermosynechococcus elongatus*” published by Korste et al. in Biochim. Biophys. Acta 1847(2015)1212–1219 [1]. The cyanobacterial multi-subunit membrane protein complex NDH-1 structurally and functionally relates to Complex I of eubacteria and mitochondria. The NDH-1 complex is mechanistically involved in respiration and cyclic electron transfer around photosystem I (PSI) as well as in a unique mechanism for inorganic carbon concentration.

**Specifications table**

**Table:** 

Subject area	Biochemistry, structural biology
More specific subject area	Nuclear magnetic resonance (NMR), protein structure calculation
Type of data	NMR distance restraints, dihedral angle restraints
How data was acquired	Multidimensional solution NMR spectroscopy
Data format	ARIA and CNS input files
Experimental factors	The NMR experiments were performed on a sample containing 0.5 mM protein in 50 mM Tris–HCl (pH 8.0), 50 mM NaCl, 10 mM deuterated dithiothreitol (DTT), and 10% D2O.
Experimental features	All NMR spectra were acquired at 298 K on BrukerBioSpin Avance-III 950, Avance-I 800, DRX-600, and DRX-500 spectrometers and processed using NMRPipe [2].
Data source location	Bochum, Germany and Osaka, Japan
Data accessibility	These restraints to calculate the structure in solution of CupS have been deposited to the Protein Data Bank (www.rcsb.org) under PDB-ID accession number **2MXA**.

**Value of the data**

• the very first NMR experimental data set to compute the three-dimensional structure of CupS in solution;
• this data set might help to elucidate the function of CupS not fully understood to date;
• this data set might serve as a reference for future studies of CupS molecular complexes.

## 1 Data

**Fig. 1:**
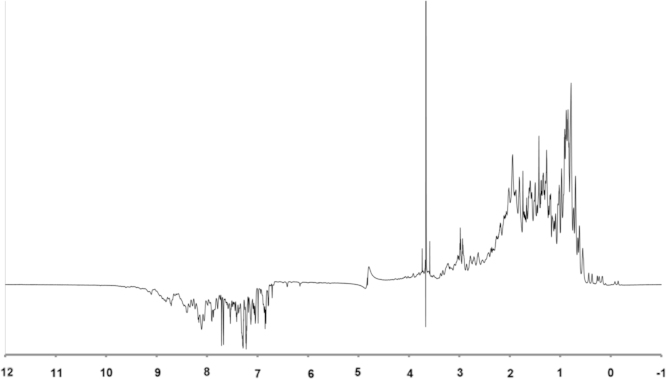
One-dimensional ^1^H NMR spectrum (with signal intensities plotted *versus* ppm values) of [^15^N]-CupS recorded on a BrukerBioSpin Avance-III 950 spectrometer at pH 8.0 and at 293 K. Proton chemical shifts in the amide region were detected without the application of decoupling pulses to ^15^N.

We have extracted a total of 2089 NOE distance restraints from three-dimensional ^15^N-edited and ^13^C-edited NOESY spectra, which were processed using NMRPipe [1], [2]. Spectra exhibit substantial chemical shift dispersion – a feature also observed for the one-dimensional ^1^H NMR spectrum of CupS (Fig. 1). In total, this data set consists of 929 intra-residual, 448 sequential, 281 medium range, and 431 long-range NOE distance restraints, supplemented by 221 NMR-derived dihedral angle restraints from TALOS+ [3]. These experimental restraints are compatible with the software suite ARIA 2.3 [4]/CNS 1.2.1 [5], [6]. NOEs were picked manually and obvious intraresidual and sequential NOEs were assigned hand-operated. ARIA2.3 [4]/CNS 1.2.1 [5], [6] and UNIO (ATNOS/CANDID) [7]/CYANA 3.0 [8] software packages were used to automatically assign the picked NOE resonances.

## 2 Experimental design, materials and methods

### 2.1 Protein purification

The cloning, expression, and the purification of isotopically enriched CupS protein has been reported recently [9].

### 2.2 NMR spectroscopy

The NMR experiments were performed on a sample containing 0.5 mM protein in 50 mM Tris–HCl (pH 8.0), 50 mM NaCl, 10 mM deuterated dithiothreitol (DTT), and 10% D2O. All NMR spectra were acquired at 298 K on BrukerBioSpin Avance-III 950, Avance-I 800, DRX-600, and DRX-500 spectrometers. The almost complete backbone and side chain chemical shift assignment has been reported recently [7]. These chemical shifts and resonance assignments have been deposited in the BioMagResBank (http://www.bmrb.wisc.edu/) under accession number 19971. In order to derive distance restraints for structure calculation, ^15^N-edited NOESY as well as ^13^C-edited NOESY spectra were recorded, each with a mixing time of 100 ms. Except for the one-dimensional ^1^H spectrum shown in Fig. 1 that was recorded with an 1-1 echo pulse sequence, most of the NMR experiments involved WATERGATE and water-flip-back methods for suppression of the water signal, except for ^13^C-edited NOESY spectra, which were measured in D_2_O-based buffer [10]. Dihedral angles were obtained from TALOS+ [3] employs C_α_ and C_β_ chemical shift values.
